# Affect and Mindfulness as Predictors of Change in Mood Disturbance, Stress Symptoms, and Quality of Life in a Community-Based Yoga Program for Cancer Survivors

**DOI:** 10.1155/2013/419496

**Published:** 2013-05-23

**Authors:** Michael J. Mackenzie, Linda E. Carlson, Panteleimon Ekkekakis, David M. Paskevich, S. Nicole Culos-Reed

**Affiliations:** ^1^Faculty of Kinesiology, University of Calgary, 2500 University Drive NW, Calgary AB, Canada T2N 1N4; ^2^Department of Kinesiology and Community Health, University of Illinois at Urbana-Champaign, USA; ^3^Department of Psychosocial Resources, Tom Baker Cancer Centre, Alberta Health Services-Cancer Care, Canada; ^4^Department of Oncology, Faculty of Medicine, University of Calgary, Canada; ^5^Department of Kinesiology, Iowa State University, USA

## Abstract

Little attention has been paid to the psychological determinants by which benefits are accrued via yoga practice in cancer-related clinical settings. Using a longitudinal multilevel modeling approach, associations between affect, mindfulness, and patient-reported mental health outcomes, including mood disturbance, stress symptoms, and health-related quality of life (HRQL), were examined in an existing seven-week yoga program for cancer survivors. Participants (*N* = 66) were assessed before and after the yoga program and at three- and six-month follow-ups. Decreases in mood disturbance and stress symptoms and improvements in HRQL were observed upon program completion. Improvements in mood disturbance and stress symptoms were maintained at the three- and six-month follow-ups. HRQL exhibited further improvement at the three-month follow-up, which was maintained at the six-month follow-up. Improvements in measures of well-being were predicted by initial positive yoga beliefs and concurrently assessed affective and mindfulness predictor variables. Previous yoga experience, affect, mindfulness, and HRQL were related to yoga practice maintenance over the course of the study.

## 1. Introduction

Receiving a cancer diagnosis, undergoing treatment, and the subsequent recovery takes a great toll on many cancer survivors. Psychosocial distress stemming from the cancer experience is a significant problem for up to half of all cancer patients, and many survivors experience lowered overall health-related quality of life (HRQL) during and following active treatment [1]. Regardless of intervention specifics, exercise enhances a variety of HRQL and psychosocial outcomes in various cancer survivor groups both during and after cancer treatment and may also help to manage the long-term side effects of treatment [2].

Within the larger field of exercise and cancer, yoga is often considered a gentle, low-intensity form of exercise [3]. The unique integration of both moving and static sequences (asana), breathing exercises (pranayama), and different meditation tools to withdraw the senses (pratyahara), concentrate the mind (dharana), and develop abilities of impartial awareness (dhyana) have all been used as means of increasing performance and recovery in both general and clinical populations [4]. These practices are routinely modified based on desired outcomes as well as participant health status [5]. Studies comparing the effects of yoga and exercise indicate that, in both healthy individuals and those with various health conditions, yoga may be as effective as more common forms of exercise, including walking, jogging, cycling, and aerobics, at improving a variety of health-related outcome measures [6]. Within clinical cancer settings, those participating in a yoga intervention compared to waitlist control groups or supportive therapy groups showed greater improvements in overall HRQL, psychological health, stress-related symptoms, sleep and fatigue indices [7–12] However, these reported improvements in cancer survivors practicing yoga have not been uniform [13].

## 2. Theory

As research in the use of yoga in cancer-related clinical settings continues to grow and yoga is integrated into cancer care, it is imperative the clinical benefits of yoga for cancer survivors are better understood. Identifying predictors that explain *how* yoga leads to clinically significant outcomes is the next step in understanding the ability of yoga to *target *desired outcomes. Recommendations from the UK Medical Research Council [14] suggest time spent examining predictors, both theoretically and practically, help to strengthen the causal chain of evidence and further refine both the interventions themselves and research design. The proposed predictors tested in the current study include affect regulation and mindfulness.

### 2.1. Affect Regulation

Positive affect is theorized to be an independent, adaptive pathway in the cancer experience [15]. Positive affect can be broadly defined as feelings associated with pleasurable experience that may elicit descriptors such as happiness, joy, contentment, and peacefulness [16]. Both baseline positive affect and the enhancement of positive affect are important components of symptom management and cancer recovery [17]. In general, exercise increases positive affect and reduces negative affect [18] and individuals tend to choose and adhere to physical activity associated with positive affective experiences [19]. In cancer-related clinical settings specifically, fostering positive affective experiences via a structured exercise program is an important target for interventions designed to facilitate postprogram exercise adherence [20].

Participation in a single yoga session has been associated with significant improvements in positive affect and reductions in negative affect, comparable to changes seen with aerobic exercise [21, 22]. Findings from a pilot randomized-controlled trial (RCT) examining the effects of a 10-week restorative yoga program in breast cancer survivors on or off treatment suggest significant benefits favoring the yoga group on positive affect, mental health, depression and spirituality outcomes [23]. Findings from a second pilot RCT examining the effects of a 12-week yoga program in breast cancer outpatients undergoing adjuvant radiotherapy reported significant improvement in positive affect, emotional function and cognitive function, and decreased negative affect in the yoga group as compared to controls [24].

#### 2.1.1. Circumplex Model of Affect

**Figure 1:**
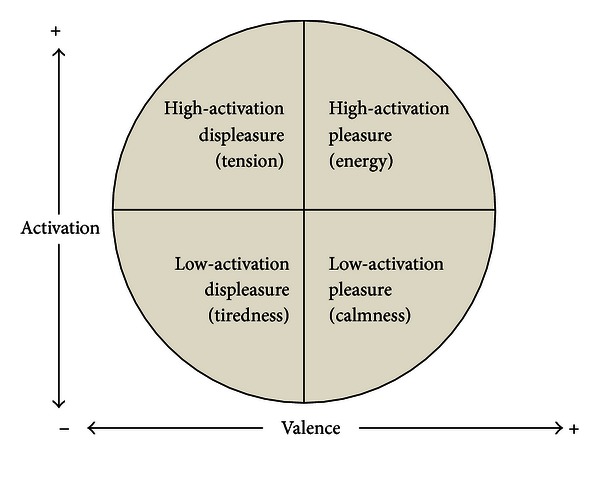
Circumplex Model of Affect.

Given the proposed importance of positive affect and exercise and yoga's influence on this phenomenon, the Circumplex Model of Affect [25] offers an encompassing yet parsimonious explanatory approach for conceptualizing and assessing changes in affect. At the heart of mood and emotion are underlying psychophysiological states experienced as simply feeling good or bad, energized or fatigued. According to the Circumplex Model, the affective domain can be represented by a circle defined by two orthogonal and bipolar dimensions: valence (pleasure versus displeasure) and perceived activation or arousal (high versus low). These two dimensions of valence and activation, termed *core affect,* underlie all affective states. Four states derived from the combination of these two dimensions can be described as high-activation pleasure (e.g., energy), low-activation pleasure (e.g., calmness), high-activation displeasure (e.g., tension), and low-activation displeasure (e.g., tiredness) [26] (see Figure 1). Based on these two dimensions and four quadrants, a host of affective responses, emotions and moods are possible. The Circumplex Model of Affect has been recommended as a conceptual framework for measuring core affect given the dimensional approach allows capture of the constituent elements of a larger range of basic affective states [27].

### 2.2. Mindfulness

Mindfulness is the systematic development of the ability to nonjudgmentally direct attention towards events in the field of consciousness in the present moment [28]. Emerging research findings suggest both trait and state mindfulness are related to affect regulation [29]. Specifically, the various extant measures of mindfulness and practices that engender higher reported mindfulness have been associated with higher reported positive affect and lower reported negative affect, perhaps through engagement of attention upon immediate experience [30].

It has been suggested yoga adds a contemplative element to exercise and can be conceptualized as “mindfulness in motion” [31]. Specifically, the practice of yoga provides an opportunity for sustained attention to physical sensations, breathing, and mental activity through progressive sequences of dynamic movements, restful postures, breathing exercises, and periods of meditative awareness [32]. However, research evidence linking yoga practice to these changes in mindfulness remains equivocal. Lengacher and colleagues [33] found minutes of yoga practice as part of the overall Mindfulness-Based Stress Reduction (MBSR) program was not significantly related to positive changes in psychosocial status and HRQL in a breast cancer survivor population. However, these findings are in contrast to the work of Carmody and Baer [34], whose research in a noncancer heterogeneous medical population found the yoga component of an MBSR program to be most strongly related to improvements of mindfulness measures, psychological symptoms and perceived stress. These findings have more recently been corroborated in a study by Sauer-Zavala and colleagues [35], which reports yoga practice was associated with greater psychological well-being independent of equivalent amounts of a supine body scan or seated meditation practice in an undergraduate student population.

#### 2.2.1. Facets of Mindfulness

Five facets of mindfulness have been described as follows: the ability to (1) observe and (2) describe experiences in the present moment while (3) acting with awareness (4) without judgment or (5) prolonged reaction [36]. In cancer-related clinical settings, work by Bränström and colleagues [37] suggest prepost changes in facets of mindfulness mediate changes in psychological outcomes both post-MBSR program and at a three- but not six-month follow-up [38]. Recent work by Garland and colleagues [39] is consistent with these findings, indicating significant relationships between increased facets of mindfulness and reductions in mood disturbance and stress symptoms.

Cancer-related distress and lower HRQL are prevalent among many cancer survivors. Exercise is effective in ameliorating this distress and improving HRQL. Yoga can be considered a low-intensity form of exercise that has been shown to independently improve HRQL, psychosocial outcomes and symptom indices. Two posited predictors of these improvements are increased positive affect and increased mindfulness.

## 3. Objectives

Given preliminary evidence that yoga practice is associated with (1) increased positive affect, (2) heightened mindfulness, and (3) improved HRQL and mental health outcomes in cancer survivors, the present study was designed with the following objectives.

### 3.1. Longitudinal Program Effects

Objective 1a: to examine changes longitudinally, before and after a yoga program and at three- and six-month follow-ups, in measures of mood disturbance (primary outcome), stress symptoms, and HRQL as well as proposed predictor variables, including affect and mindfulness. Objective 1b: to examine whether improvements in mood disturbance, stress symptoms and HRQL were associated with concurrent changes in proposed predictor variables, including affect and mindfulness.

### 3.2. Yoga Practice Maintenance

Objective 2a: to examine whether participants maintain their yoga practice longitudinally, before and after a yoga program and at three- and six-month follow-ups. Objective 2b: to examine whether maintenance of yoga practice was associated with mood disturbance, stress symptoms and HRQL as well as proposed predictor variables, including affect and mindfulness.

## 4. Methods

### 4.1. Participants

Ethical approval was obtained from the Conjoint Health Research Ethics Board of the University of Calgary/Alberta Health Services. Program participants were comprised of a heterogeneous group of cancer survivors enrolled in the ongoing “Yoga Thrive: Therapeutic Yoga for Cancer Survivors” program. Participants were eligible for study inclusion if they were (1) aged 18 years or older and (2) had received a cancer diagnosis at any time in the past. Previous participation in the Yoga Thrive program was not an exclusion criterion but was evaluated as part of the study. Participants were informed of the research study at the time of class registration, either via telephone or online. Those that indicated interest in participating in the research study were contacted by the study coordinator. Baseline, postprogram, three- and six-month follow-up surveys were completed online.

### 4.2. Program

The Yoga Thrive program is a research-based, therapeutic yoga program for cancer survivors and their support persons. This gentle seven-week yoga program is based on contemporary yoga practices modified for cancer survivors. Details of the program have been previously described [40, 41]. The DVD “Yoga Thrive: Therapeutic Yoga for Cancer Survivors” also includes the entire 7-week program. A typical 75-minute class was as follows.

 0–10 minutes: gentle breathing and movement, laying supine, with legs flexed at the hip and supported by a wall.
 10–60 minutes: 6–10 modified yoga postures/sequences consisting of gentle stretching and strengthening exercises with attention to breath and bodily sensations. Participants progressed from a series of sitting and kneeling postures to standing postures, including stronger postures, balance work and forward bends, before returning to the floor for a series of supine postures.
 60–75 minutes: guided supine meditation with attention placed on both breathing and bodily sensations. The yoga classes became progressively more challenging over the seven-week course.

### 4.3. Recruitment

Power calculations were carried out using GPower 3.1 [42]. The sample size/power calculation was based on a clinically significant [43] change in score before-after program on the Profile of Mood States (POMS) Total Mood Disturbance (TMD) score as the primary outcome (Cohen's *d* = .50). With an *α* of .05 and power of .80, a minimum of 34 participants were to be recruited from the Yoga Thrive program.

### 4.4. Instruments

#### 4.4.1. Baseline 

*(1) Demographics*. Demographic information included self-reported age, education, marital status, and current employment status. Medical history included self-reported cancer diagnosis, date of diagnosis and type(s) of cancer treatment.

*(2) Beliefs about Yoga Scale (BAYS)*. The BAYS [44] is an 11-item self-report measure developed to examine common positive and negative beliefs about yoga in order to help understand participant expectations related to yoga (baseline *α* = .78).

#### 4.4.2. Longitudinal Effects (Objectives 1a and 1b) 

*(1) Godin Leisure Time Exercise Questionnaire (GLTEQ).* The GLTEQ [45] was used to assess physical activity levels. The Leisure Score Index (LSI) subscale of the GLTEQ contains three questions that assess the frequency of mild, moderate, and strenuous physical activity performed for at least 15 minutes duration in a typical week within the past month. In addition, a weekly total of moderate-to-vigorous physical activity can be computed from the GLTEQ (baseline *α*: LSI = .88; moderate-to-vigorous physical activity = .86).

*(2) Activation-Deactivation Adjective Check List (AD ACL).* The AD ACL [46] is a 20-item measure that has been utilized previously to map the four quadrants of circumplex affective space [47]. The energy pole is theorized to map the high-activation pleasure quadrant of the circumplex, tension maps the high-activation displeasure quadrant, tiredness maps the low-activation displeasure quadrant, and calmness maps the low-activation pleasure quadrant [26] (baseline *α*: energy = .88; tension = .79; tiredness = .84; calmness = .80).

*(3) Five*-*Facet Mindfulness Questionnaire (FFMQ)*. The FFMQ [48] is a 39-item scale designed to measure five factors that represent elements of trait mindfulness as it is currently conceptualized. The five facets are observing, describing, acting with awareness, nonjudging of, and nonreaction to inner experience. Higher scores indicate higher levels of mindfulness (baseline *α*: observing = .85; describing = .87; acting with awareness = .89; nonjudgment = .91; nonreaction = .88).

*(4) Profile of Mood States-Short Form (POMS-SF)*. The abbreviated POMS-SF [49] is a 37-item scale designed to assess six distinct mood states (tension, depression, anger, vigor, fatigue, and confusion) over a one-week period. The instrument also provides a total mood disturbance score by summing the five negative mood state scores and subtracting the one positive score (vigor). Only the POMS-SF total score was used.

*(5) Calgary Symptoms of Stress Inventory (C-SOSI)*. The abbreviated C-SOSI [50] is a 56-item scale designed to assess physical, psychological, and behavioral responses to stressful situations. The instrument provides a total stress score as well as eight subscale scores (depression, anger, muscle tension, cardiopulmonary arousal, sympathetic arousal, neurological/GI, cognitive disorganization, and upper respiratory symptoms). Higher scores indicate higher reported levels of stress symptoms. Only the C-SOSI total stress score was used.

*(6) Functional Assessment of Cancer Therapy-General Version (FACT-G).* FACT-G (version 4) [51] is a 27-item questionnaire that measures HRQL. The instrument provides a total HRQL score as well as four subscale scores (physical, social, emotional, and functional). Higher scores indicate higher reported HRQL. Only the FACT-G total score was used.

#### 4.4.3. Yoga Practice Maintenance (Objectives 2a and 2b)

Participants were asked to report weekly frequency of ongoing yoga practice via the Yoga Thrive program, community-based yoga programs, home yoga practice or combinations thereof.

### 4.5. Data Analysis

All data analyses were conducted using IBM SPSS version 19. Demographics and medical history were described using frequency and descriptive statistics to characterize study participants. Statistical analyses were conducted on the entire sample from baseline (*N* = 66).

#### 4.5.1. Longitudinal Effects (Objectives 1a and 1b)

Multilevel modeling provides a powerful, flexible framework for analyzing nested data structures longitudinally and how change over time in one variable may be related to change over time in other variables [52]. Put in another way, multilevel models allow estimation of both growth and assessment of predictors of differences in that growth for any given outcome of interest. Multilevel models are appropriate for analyzing data with dependent observations (such as within-subject repeated measures) [53]. In addition, multilevel models accommodate all available data, retaining cases for which missing data are present, and provide a valid analysis when data are assumed missing at random [54]. This inclusion of all available data is particularly useful when conducting intention-to-treat (ITT) analyses [55].

In the current analyses two sets of multilevel models were employed. Firstly, estimated marginal means models were created to assess overall change in outcome, predictor, and continuous covariates longitudinally, pre-post and at three-and six-month follow-ups (objective 1a). Secondly, multilevel regression analyses were conducted to assess concurrent associations over time between mood disturbance, stress symptoms and HRQL, proposed predictor variables including affect and mindfulness, and covariates including demographics, time since cancer diagnosis, physical activity, beliefs about yoga, previous yoga experience, and yoga program attendance (objective 1b). Correlations among observations from the same individual were modeled using an unstructured covariance matrix across all time points. By fitting each individual growth trajectory to a specific parametric model, the overall trajectory of the study sample was obtained and allowed for further investigation of whether differences in growth parameters were related to other predictor variables [56].

In each multilevel regression model, time was measured continuously and included linear, quadratic, and cubic terms as appropriate [56]. The time variable was centered at initial status; therefore the intercept of the regression model was interpreted as participant reports of the outcome variable at baseline. To enhance interpretability of model intercept parameters, all predictor variables were grand-mean centered to allow for inference of average predictor effects [57]. Relationships between predictor and outcome variables were assumed to be constant throughout the study if interaction terms were not significant.

All models were tested step by step. An initial unconditional model was developed for each outcome variable, followed by unconditional growth models. Based on these growth models, predictor variables were tested individually for main effects and for interaction effects with each time term. Significant predictors and their time interactions, if significant, were then tested together as part of their respective overall scale. A final trimmed conditional growth model was developed by entering all significant predictors and their interactions to test overall prediction of outcome variables across time (exclusion *P* > .1). This method of model development has proven robust with smaller sample sizes and ensures these models do not tax the “carrying-capacity” of the dataset [58].

*(1) Clinical Significance (Objectives 1a and 1b).* In the estimated marginal means models (objective 1a), clinical significance, a marker of program effectiveness, was calculated using Cohen's *d*, a distribution-based method, for each outcome variable between baseline and (a) postprogram (8 weeks), (b) three-month, and (c) six-month follow-up (*Tx* − *T*_1_)/(*T*_1_SE∗√*N*). These effects were interpreted using Cohen's interpretation of .20 as a small effect .50 as a moderate effect and .80 as a large effect [59]. In the multilevel regression analyses (objective 1b), pseudo *R* ^2^ statistics for each model were calculated as unconditional model residual variance − trimmed model residual variance/unconditional model residual variance. These statistics indicate the proportional reduction in residual variance (error) between the unconditional and trimmed model and provide an estimate of effect size similar to traditional ordinary least squares (OLS) regression [60]. These effects can be interpreted using Cohen's criteria of .02 as a small effect .13 as a moderate effect and .26 as a large effect [59].

#### 4.5.2. Yoga Practice Maintenance (Objectives 2a and 2b)

Associations of predictor and outcome variables with program maintenance (ongoing yoga participation in either the yoga program for cancer survivors, community-based yoga programming, or engaging in home practice) were assessed via logistic generalized estimating equations (GEE) [61]. In this context, GEE take into consideration the within-subject relationships between predictor and outcome variables. Yoga maintenance was dichotomized as either 0—no yoga or 1—ongoing yoga. To determine whether there was a difference between those who practiced yoga and those who did not at each time point, an initial estimated marginal means model with no predictors was run (objective 2a). Logistic GEE were then run to determine associations between yoga practice and predictor variables (objective 2b). Covariates for demographics, time since diagnosis, beliefs about yoga, previous yoga experience and yoga program attendance were entered individually as main effects, followed by physical activity, affect, mindfulness and health outcomes. A final trimmed model was developed by entering all significant predictors and their interactions to test overall prediction of yoga practice maintenance across time (exclusion *P* > .1).

## 5. Results

### 5.1. Demographics

**Figure 2:**
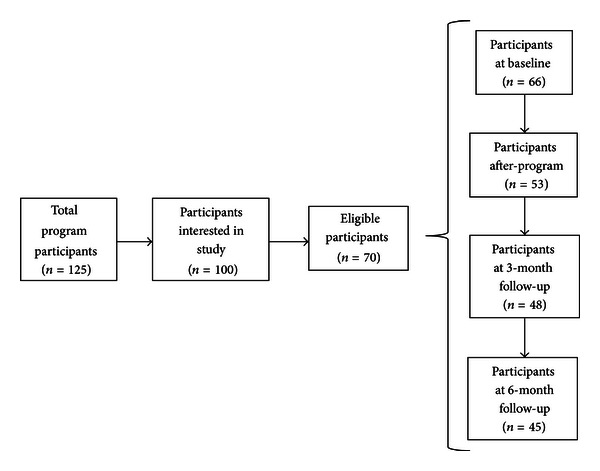
Participant recruitment flow diagram.

70 participants were eligible for the current study and 66 completed baseline measures used in the current analyses (see Figure 2). The average participant was approximately 53 years of age. The study sample was 90% female. The sample was comprised primarily of participants had received a breast cancer diagnosis (62.1%). The two other most common diagnoses were lymphoma (10.6%) and colorectal (7.6%). The majority of participants had been diagnosed stages II-III (59.1%) approximately two years prior to study enrollment. Most participants were married (66.7%), highly educated (54.5% had completed university/college), and affluent (60.6% had a combined household income >$80,000 per annum). Many participants (39.4%) had returned to work fulltime. Participants attended an average of five of the seven yoga sessions (see Table 1).

### 5.2. Longitudinal Program Effects (Objectives 1a and 1b)

Estimated marginal means models were computed for covariates including age, time since diagnosis, physical activity, and previous yoga experience, predictor variables including affect and mindfulness, and outcome variables including mood disturbance, stress symptoms, and HRQL (objective 1a).

#### 5.2.1. Covariate Predictors

Participants had already completed Yoga Thrive 1.46 times prior to study enrollment. Most participants reported completing the Yoga Thrive program at eight-weeks, and had completed the program an additional time by the six month follow-up [*F*(3,147.14) = 74.32, *P* < .001]. Small significant increases from baseline in moderate-to-vigorous PA were observed at the three- and six-month follow-ups. A small significant increase in total physical activity (LSI) was also observed over time [*F*(3,143.52) = 3.28, *P* = .023] (see Table 2).

#### 5.2.2. Affect

Small increases in energy were observed post -program that became more pronounced at the three- and six-month follow-ups [*F*(3,148.73) = 11.98, *P* < .001]. Small decreases in tiredness after-program were not maintained at the 3-month follow-up but again improved at the 6-month follow-up [*F*(3,151.01) = 5.99, *P* < .001]. Small decreases in tension were observed after-program, and were not maintained at the three-month follow-up, but again improved at the six-month follow-up [*F*(3,145.19) = 4.53, *P* = .005]. There were no changes in patient-reported calmness over time (see Table 2).

#### 5.2.3. Mindfulness

No changes in participants' observational skills or ability to describe events in the field of consciousness were observed throughout the program. Moderate increases in participants' ability to act with awareness were observed after-program that were maintained at the three- and six-month follow-ups [*F*(3,138.05) = 9.46, *P* < .001]. Small increases in participants' ability to be nonjudgmental of inner experience were observed after-program that were maintained at the three- and six-month follow-up [*F*(3,138.92) = 3.74, *P* = .013]. No significant changes in nonreaction were observed after-program but small significant improvements were observed at the three- and six-month follow-up [*F*(3,138.90) = 4.25, *P* = .007] (see Table 2).

#### 5.2.4. Mood Disturbance, Stress Symptoms and Quality of Life

Decreases in mood disturbance were moderate after program. Reductions in mood disturbance were not maintained at the three-month follow-up but improved to postprogram values at the six-month follow-up [*F*(3,139.56) = 5.72, *P* < .001]. A moderate decrease in stress symptoms was observed from baseline to after program that was maintained at both the three- and six-month follow-ups [*F*(3,139.12) = 12.21, *P* < .001]. A small statistically significant improvement in HRQL was observed after program. However, HRQL significantly improved at the three-month follow-up and was maintained at the six-month follow-up [*F*(3,137.33) = 7.93, *P* < .001] (see Table 2).

Follow-up multilevel regression analyses were conducted to explore the associations between mood disturbance, stress symptoms, and HRQL, predictor variables including affect and mindfulness, and covariates including age, time since diagnosis, physical activity, previous yoga experience, and yoga beliefs (objective 1b). 

#### 5.2.5. Mood Disturbance

Significant linear, quadratic, and cubic effects were observed for time. Those with more positive beliefs in yoga at baseline reported lower mood disturbance at all time points. Those who reported higher levels of overall PA, higher energy, and higher levels of the ability to act with awareness and be nonreactive to inner experience also reported lower mood disturbance. Those who reported greater tiredness reported greater mood disturbance. The Pseudo *R* ^2^ value suggests inclusion of these variables reduced error in predicting mood disturbance by 31% (see Table 3). 

#### 5.2.6. Stress Symptoms

There were significant linear and quadratic effects of time, indicating an initial decline in stress symptoms that slowed over time. Higher positive beliefs about yoga at baseline were associated with lower symptoms of stress. Those who reported higher moderate-to-vigorous PA and a greater ability to act with awareness and be nonjudgmental of inner experience concurrently reported lower stress symptom scores. Higher tension was significantly associated with higher stress symptoms. The Pseudo *R* ^2^ value suggests inclusion of these variables reduced error in predicting stress symptoms by 40% (see Table 3).

#### 5.2.7. Quality of Life

A non-significant linear increase in HRQL was observed over time. Higher baseline beliefs about yoga were associated with higher HRQL. Those who reported higher overall PA, energy, and the ability to be nonjudgmental of inner experience reported higher HRQL. Those who reported high levels of tension reported lower levels of HRQL. The Pseudo *R* ^2^ value suggests inclusion of these predictor variables reduced error in predicting HRQL by 29% (see Table 3).

### 5.3. Yoga Practice Maintenance (Objectives 2a and 2b)

Longitudinal GEE logistic estimated marginal means models were conducted to examine yoga practice before and after the yoga program and at three- and six-month follow-ups (objective 2a). Upon initiation of the current study, 48% (32 participants) reported previous yoga practice experience either through the Yoga Thrive program, community classes, home practice, or combinations thereof. At the end of the seven-week yoga program, 96% were still practicing yoga in various settings, a significant increase of 48% from baseline. At the three-month follow-up yoga practice had dropped, with 69% of participants were still practicing yoga in various settings, a significant 20% increase from baseline. At the six-month follow-up there was a slight increase in participation rates, with 76% of participants reporting continued yoga practice in various settings, a significant 27% increase from baseline (see Table 4).

Follow-up GEE logistic regression analyses were performed to examine what participant characteristics predicted ongoing yoga practice (objective 2b). In examining associations between yoga practice at each time point and predictor variables, there was an initial positive linear effect for time, reflecting the increased rate of participants reporting yoga practice before-after program. The quadratic trend reflects a significant decline postintervention completion in yoga practice at three- and six-months. Those who reported more frequent participation in the Yoga Thrive program at each time had a greater chance of continuing their yoga practice for each time they completed the Yoga Thrive program. In addition, those who reported higher energy at each time point were more likely to maintain a yoga practice, as were those who reported a greater ability to be nonreactive to inner experience. Finally, those with higher self-reported HRQL at each time point had a greater chance of continuing yoga practice (see Table 5).

## 6. Discussion

Despite an emerging body of evidence highlighting the benefits of yoga for cancer survivors, little work has been done to bridge these clinical findings with theoretical predictors of change. The current research sought to bridge this gap between determining clinical benefits and describing predictors of these improvements in mood disturbance, stress symptoms and HRQL. Using the Circumplex Model of Affect as it has been applied in exercise settings [62] and emerging work examining the mechanisms of mindfulness [30, 36, 63] and yoga [64–66], a series of multilevel models were developed to examine (1) longitudinal changes in mood disturbance, stress symptoms and HRQL, predictor variables including affect and mindfulness, and whether these variables predicted change in these outcomes (objectives 1a and 1b); and (2) maintenance of yoga practice and whether maintenance could be anticipated by the aforementioned outcome and predictor variables (objectives 2a and 2b).

### 6.1. Longitudinal Program Effects (Objectives 1a and 1b)

#### 6.1.1. Affect

Current study results suggest a linear increase in energy over time. These findings concur with research in the area of aerobic training [18] which suggest positive improvements in high-activation positive affect (e.g., energy) over time when exercise is of lower intensity and duration. Given the burden of fatigue in cancer survivors, improvements in energy are important and corroborate corollary improvements in fatigue indices in both the exercise and cancer [67–69], mindfulness [70, 71] and yoga literature [9].

#### 6.1.2. Mindfulness

Longitudinal findings suggest participants reported improved ability to act with awareness, a mindfulness facet closely related to one's ability to concentrate [36]. Participants also reported increased nonjudging of inner experience, the taking of an impartial stance to thoughts and feelings, and nonreaction to inner experience, the ability to let thoughts and emotions come and go without having to act upon them. Research suggests the mindfulness facets of acting with awareness, nonjudgment, and nonreaction are most closely tied to decreased psychological distress [48]. Similar improvements in these mindfulness facets were also reported by Garland et al. [39] and Bränström et al. [37, 38] although effects reported in these other studies were of a larger magnitude and included improvements in the other facets of mindfulness as well. One potential explanation is that yoga practice may differ from formal mindfulness meditation training in preferentially developing skills of acting with awareness, nonjudging, and nonreactivity but not similarly help to develop other mindfulness skills of observing and describing one's experience.

#### 6.1.3. Mood Disturbance, Stress Symptoms, and Quality of Life

Baseline positive yoga beliefs were consistently associated with lower mood disturbance, stress symptoms, and higher HRQL at all time points. Sohl et al. [44] suggest baseline yoga beliefs are intrinsic to both initial yoga program engagement and reported health outcomes. This speaks to the importance of patient preferences in choosing treatments and the role that positive expectancies play in driving improvements [72]. In addition, those who reported higher physical activity concurrently reported lower mood disturbance, stress symptoms and higher HRQL at all time-points. These findings reflect research suggesting the effects of exercise on mental health and HRQL in cancer survivors [67, 73]. 

The present research ascribed to Russell's (2003) thesis that when affective dimensions are examined longitudinally, they are no longer tied to a specific context as in acute exercise settings, where the temporal proximity to an exercise session is evident. Rather, these measures become reflective of what Russell (2003) has termed prolonged, objectless *core affect* [25]. It could be argued that including both the POMS-SF and ADACL created measurement redundancies, as both include items that tap the construct of mood. However, the two measures are fundamentally different from a conceptual standpoint. The POMS-SF has been used to measure mood reflecting discrete, largely negative, mood states. On the other hand, the ADACL has been used to tap the two basic affective dimensions of valence and activation, which, when combined, can theoretically provide a representation of the entire affective domain, including positive and negative, as well as high- and low-activation variants of affective experience. Given that yoga theoretically may generate a range of affective states, including both low-activation pleasure (calmness) and high-activation pleasure (energy), and given that cancer survivors experience both low-activation displeasure (tiredness) and high-activation displeasure (tension), it behooves researchers to utilize measures that capture the full range of affective experiences. Furthermore, dimensional measures of affective states may constitute more categorical mood states, including the mood states captured by the POMS-SF [15, 74, 75].

Higher reported ability to act with awareness was associated with lower mood disturbance and stress symptoms at all times. Higher reported nonjudgment of inner experiences was related to lower stress symptoms and higher HRQL. These improvements may reflect an accepting attention strategy in which participants find alternate ways to respond to stressful situations [30]. These findings are similar to the study by Garland et al. [39], in which both acting with awareness and being nonjudgmental of inner experience were correlated with reduced stress and mood disturbance. Higher non-reactivity to inner experience was related to lower mood disturbance. Research with experienced meditators and non-meditators suggests the ability to be nonreactive to inner experience is highly related to meditation experience and is most likely to mediate psychological health outcomes [76].

The present research is congruent with previous work suggesting yoga's effectiveness in reducing psychological distress and improving HRQL in cancer survivors [7, 9]. Continued significant changes in all three measured health outcomes were observed, despite the fact that 48% of participants at baseline were already yoga practitioners and had arguably already derived initial program benefits. Findings suggest improvements in health outcomes are associated with beliefs about yoga, physical activity, affective dimensions of energy, tension and tiredness, and facets of mindfulness, including the ability to act with awareness, be nonjudgmental of and nonreactive to inner experience. Associations between positive affective states and mindfulness are becoming clearer as state and trait measures of both constructs have been found to be highly related [29].

### 6.2. Yoga Practice Maintenance (Objectives 2a and 2b)

Previous participation in the Yoga Thrive program was a significant predictor of yoga practice maintenance at all time points. This finding is similar to Speed-Andrews and colleagues' finding that yoga experience over the past year was significantly related to current yoga adherence in a cancer setting [77]. Positive experiences and increased well-being derived from yoga classes are suggested mechanisms for this increased program adherence [78].

Those reporting higher energy at each time point were also more likely to continue yoga practice. This relationship between positive affect and adherence has been widely reported in the literature [18, 19]. It is hypothesized the positive appraisal of physical activity as pleasurable is likely to bolster participant self-efficacy and lead to increased adherence and maintenance [79]. Recent American College of Sports Medicine (ACSM) guidelines also suggest positive affect may be an important determinant of exercise adherence [80].

Results suggesting those higher in non-reactivity were more likely to adhere to yoga practice are also supported in the literature. Nonreaction refers specifically to the ability to remain calm in the face of distressing thoughts and emotions. This ability to be nonreactive to inner experience has salutary effects and seems to be a key facet of mindfulness as well as one of the facets most affected by contemplative training [76]. Research by Ulmer and colleagues [81] suggests those with higher mindfulness scores are less reactive and better able to accurately appraise and respond to stressors that may impact routine activities, including yoga practice.

Results from the current study suggest a reciprocal relationship in which higher HRQL is associated with yoga practice maintenance. Higher HRQL has been associated with increasing individual self-efficacy to adhere and maintain exercise programs [79]. Maintenance of pre-cancer diagnosis physical activity levels and bodyweight is associated with better HRQL after breast cancer [82] and those who report meeting physical activity requirements in general report higher HRQL across cancer diagnoses [83].

Taken together these results suggest previous yoga experience coupled with higher energy, a greater ability to be nonreactive to inner experience and higher HRQL interact to improve the likelihood participants will maintain yoga practice, arguably further engendering the benefits of these practices. If affect and mindfulness are both associated with improved mental health outcomes and HRQL and cited as leading to increased program adherence, it behooves clinicians to consider developing approaches that focus more exclusively on the inherent psychological benefits of exercise, including yoga [62, 80].

### 6.3. Limitations

The use of an observational research design, including 48% of study participants who had already completed the program, added complexity to the current analyses. Without a comparison group, it is impossible to determine whether positive program effects were related to the yoga program, or due to the simple passage of time, among other factors. Also, given the high degree of previous yoga experience within the study sample, these findings are not generalizable to a general cancer survivor population. Despite these limitations, a comparison group was not utilized. Rather, the researchers elected to conduct the research in a “real-world” clinical setting given the Yoga Thrive programs' broad community-based implementation and the researchers' stated goal of identifying and developing theory to examine likely predictors of change in participant-reported outcomes [14]. However, as yoga program specific predictors of change are posited, subsequent research designs should include comparative effectiveness studies in which pragmatic trials use real-world comparison conditions rather than attention or waitlist controls using uniformly yoga naïve participants [84]. An additional limitation of the current study is the lack of a measure of home practice. Current work by Ross et al. [85] suggests the addition of home practice predicts mindfulness, subjective well-being, fatigue, sleep, and a variety of behavioral outcomes. In addition, frequency of yoga home practice was a better predictor of health than either total years of practice or class frequency. Given these findings, the thorough assessment of yoga practice outside of class time is imperative. Additional limitations include the self-report nature of all outcomes. While distribution-based markers of clinical significance have been introduced for these outcomes, future research would be better served by including more objective anchor-based methodology as well as supportive biomarkers of change [86]. While the study was powered on detecting a 0.50 difference on the POMS in the estimated marginal means models it was not powered for the multilevel regression analyses. However, research suggests samples of 50 study participants may be sufficient and show little bias in the regression coefficients [87]. In addition, no corrections were made for multiple comparisons. Therefore, care must be exercised in the interpretation of statistical significance due to the potential for false-positive (Type I error) findings.

## 7. Conclusion

Before yoga can be broadly applied within oncology, carefully designed and executed research that convincingly evaluates not only the effectiveness of yoga in clinical settings but also posits potential predictors of program outcomes are required. Despite the aforementioned limitations, longitudinal multilevel analyses allowed for the integration of theory to develop a novel research design able to examine mental health outcomes, contextual factors, and interactions thereof, within an existing community-based yoga program for cancer survivors. This combined knowledge can be translated directly back into the community to further develop innovative yoga programs and best practices with the express aim of improving mental health and HRQL in cancer survivors, soothing both mind and body.

## Figures and Tables

**Table 1:** Demographics.

Baseline (*N* = 66)	
Age, years (SD)	52.88 (8.11)
Time since diagnosis, months (SD)	23.93 (21.93)
Program attendance, number of sessions (SD)	5.08 (1.94)

	*N* (%)

Cancer diagnosis: breast	
Breast	41 (62.1%)
Lymphoma	7 (10.6%)
Colorectal	5 (7.6%)
Cancer stage	
II	22 (33.3%)
III	17 (25.8%)
Gender: female	60 (90.9%)
Marital status: married/common-law	44 (66.7%)
Education level: completed university/college	36 (54.5%)
Annual household income: >$80,000	40 (60.6%)
Employment status: full time	26 (39.4%)

**Table 2:** Longitudinal estimated marginal means model.

Measures	Baseline	8-weeks after			3-month follow-up			6-month follow-up		
	*T*1 (*N* = 66)	*T*2 (*n* = 53)			*T*3 (*n* = 48)			*T*4 (*n* = 45)		
	Mean (SE)	Mean (SE)	*T*2 − *T*1 SMD		Mean (SE)	*T*3 − *T*1 SMD		Mean (SE)	*T*4 − *T*1 SMD	
			*P*	*d*		*P*	*d*		*P*	*d*
Outcome variables										

Mood disturbance										
POMS-SF total	20.20 (2.46)	11.58 (2.61)	.000	−0.47	15.01 (2.67)	.036	−0.27	11.63 (2.74)	.001	−0.44
Stress symptoms										
C-SOSI total	59.08 (3.10)	47.41 (3.26)	.000	−0.63	46.78 (3.31)	.000	−0.64	49.11 (3.35)	.000	−0.51
Quality of life										
FACT-G total	73.51 (1.98)	76.71 (2.06)	.017	0.30	78.97 (2.09)	.000	0.50	79.66 (2.12)	.000	0.54

Predictor variables										

Program experience (Yoga Thrive)										
Enrolment	1.46 (0.29)	2.45 (0.29)	.000	1.00	3.02 (0.30)	.000	1.37	3.49 (0.30)	.000	1.73
Physical activity										
LSI	21.70 (2.03)	23.39 (2.18)	.388	0.11	25.39 (2.22)	.066	0.23	27.83 (2.26)	.003	0.38
GLTEQ	92.94 (13.64)	96.50 (14.52)	.788	0.03	121.74 (15.27)	.042	0.26	120.48 (15.48)	.055	0.24
Affect										
Energy	9.53 (0.52)	10.88 (0.57)	.022	0.29	11.86 (0.59)	.000	0.48	13.11 (0.60)	.000	0.72
Tiredness	11.60 (0.54)	10.16 (0.59)	.023	−0.29	10.62 (0.62)	.136	−0.19	8.82 (0.63)	.000	−0.52
Tension	9.35 (0.43)	7.66 (0.47)	.001	−0.43	8.47 (0.49)	.084	−0.23	7.99 (0.50)	.009	−0.33
Calmness	12.77 (0.43)	13.77 (0.47)	.054	0.24	13.27 (0.49)	.350	0.12	13.23 (0.50)	.399	0.10
Mindfulness										
Observe	24.92 (0.79)	25.81 (0.85)	.244	0.15	26.08 (0.87)	.139	0.18	27.13 (0.90)	.007	0.34
Describe	23.04 (0.85)	23.86 (0.90)	.243	0.16	23.20 (0.92)	.821	0.03	24.12 (0.93)	.151	0.18
Act w/Awareness	18.54 (0.69)	20.40 (0.73)	.000	0.46	20.31 (0.74)	.001	0.42	21.23 (0.75)	.000	0.62
Nonjudgment	20.35 (0.84)	22.47 (0.90)	.006	0.35	21.96 (0.92)	.041	0.34	22.65 (0.94)	.005	0.36
Nonreaction	19.92 (0.63)	20.82 (0.67)	.082	0.22	21.61 (0.68)	.002	0.40	21.51 (0.69)	.004	0.36

*T*: time, SMD: standard mean difference, SE: standard error, *P*: significance, and *d*: Cohen's *d*.

**Table 3:** Longitudinal multilevel regression model.

Predictor	Mood disturbance (POMS-SF total)				Stress symptoms (C-SOSI total)				Quality of life (FACT-G total)			
	Est. (SE)	df	*t*	*P*	Est. (SE)	df	*t*	*P*	Est. (SE)	df	*t*	*P*
Intercept	16.98 (2.10)	76.35	8.09	.000	54.89 (2.40)	69.19	22.87	.000	76.95 (1.49)	60.94	51.53	.000
Time												
Linear Time	−5.36 (2.22)	91.15	−2.41	.018	−3.90 (0.97)	108.75	−4.04	.000	0.27 (0.16)	60.05	1.65	.104
Time^2^	1.59 (0.74)	86.47	2.16	.033	0.41 (0.11)	90.10	3.62	.000	—	—	—	—
Time^3^	−0.12 (0.06)	85.68	−1.96	.053	—	—	—	—	—	—	—	—
Predictors												
Beliefs about Yoga	−0.57 (0.22)	52.55	−2.63	.011	−0.79 (0.27)	50.62	−2.87	.006	0.42 (0.19)	57.19	2.23	.030
Physical activity												
Leisure score index	−0.17 (0.08)	161.15	−2.17	.032	—	—	—	—	0.14 (0.05)	173.61	2.86	.005
Mod.-to-vigorous PA	—	—	—	—	−0.03 (0.01)	174.50	−2.10	.037	—	—	—	—
Affect												
Energy	−0.67 (0.34)	178.62	−1.96	.052	—	—	—	—	0.44 (0.17)	158.34	2.62	.010
Tension	—	—	—	—	1.35 (0.33)	158.74	4.07	.000	−0.81 (0.19)	150.36	−4.27	.000
Tiredness	0.75 (0.31)	176.96	2.45	.015	—	—	—	—	—	—	—	—
Mindfulness												
Act w/awareness	−0.58 (0.25)	144.80	−2.33	.021	−0.67 (0.29)	174.16	−2.29	.023	—	—	—	—
Nonjudgment	—	—	—	—	−0.76 (0.24)	185.89	−3.20	.002	0.56 (0.12)	180.77	4.71	.000
Nonreaction	−0.56 (0.26)	140.04	−2.13	.035	—	—	—	—	—	—	—	—

Pseudo R^2^	Mood disturbance = 0.31				Stress symptoms = 0.40				Quality of life = 0.29			

Variables excluded (*P* > .1): age, time since diagnosis, previous Yoga Thrive program experience, current Yoga Thrive class attendance, calmness (ADACL), observe and describe (FFMQ), Est.: estimate, df: degrees of freedom, and *P*: significance.

**Table 4:** Yoga practice maintenance estimated marginal means model.

Measures	Baseline	8-weeks after			3-month follow-up			6-month follow-up		
	*T*1 (*N* = 66)	*T*2 (*n* = 53)			*T*3 (*n* = 48)			*T*4 (*n* = 45)		
	Mean (SE)	Mean (SE)	*T*2 − *T*1 SMD		Mean (SE)	*T*3 − *T*1 SMD		Mean (SE)	*T*4 − *T*1 SMD	
			*P*	*d*		*P*	*d*		*P*	*d*
Adherence	0.48 (0.06)	0.96 (0.03)	.000	0.98	0.69 (0.07)	.008	0.41	0.76 (0.06)	.002	0.55

*T*: time, SMD: standard mean difference, SE: standard error, *P*: significance, and *d*: Cohen's *d*.

**Table 5:** Predictors of Yoga practice maintenance.

Parameter	*B* (SE)	Wald χ^2^	Odds ratio (95% CI)	*P*
(Intercept)	1.50 (0.38)	15.77	4.50 (2.14, 9.45)	.000
Time	0.63 (0.23)	7.11	1.87 (1.18. 2.96)	.008
Time^2^	−0.09 (0.03)	10.83	0.91 (0.86, 0.96)	.001
Previous participation in yoga thrive	0.63 (0.22)	8.42	1.88 (1.23, 2.87)	.004
Energy	0.13 (0.06)	4.94	1.14 (1.02, 1.27)	.026
Nonreaction to inner experience	0.08 (0.03)	5.86	1.08 (1.02, 1.16)	.015
Quality of life	0.03 (0.01)	4.23	1.03 (1.00, 1.06)	.040

Excluded variables (*P* > .1): age, time since diagnosis, yoga beliefs, attendance, physical activity, tension, tiredness, calmness (ADACL), observe, describe, act with awareness, nonjudgment (FFMQ), mood disturbance (POMS-SF), and stress symptoms (C-SOSI). *B*: estimate, SE: standard error, and *P*: significance.
